# Antimicrobial resistance of commensal and extended-spectrum ß-lactamase/AmpC-producing *Escherichia coli* in organic meat chicken farms

**DOI:** 10.1016/j.psj.2026.106559

**Published:** 2026-01-30

**Authors:** Anna Maria Korves-Wilm, Mirjam Grobbel, Bernd-Alois Tenhagen

**Affiliations:** Department Biological Safety, German Federal Institute for Risk Assessment, Berlin, Germany

**Keywords:** antimicrobial resistance, ESBL, organic, broiler, *Escherichia coli*

## Abstract

We examined the antimicrobial resistance of commensal *Escherichia coli* and occurrence of extended-spectrum beta-lactamase (ESBL)-/AmpC-producing *E. coli* in organic meat chicken flocks of three different fattening types using a longitudinal study design. Fourteen German small scale meat chicken farms fattening either slow-growing broiler, dual-purpose cockerels or male layer hybrids were sampled between 2023 and 2025. Throughout the fattening period, four consecutive flocks per farm were sampled five times each. Three isolates per sampling time point were picked from MacConkey agar (MCA). Additionally, MCA + 1mg/L cefotaxime (MCA+CTX) was used to selectively screen for ESBL-/AmpC-producing *E. coli*. In total, 696 commensal *E. coli* from MCA and 51 ESBL-/AmpC-producing *E. coli* from MCA+CTX were isolated. Antimicrobial resistance was determined using broth microdilution and minimum inhibitory concentrations were evaluated using epidemiological cut-off values. Throughout the fattening period, most commensal *E. coli* were susceptible in slow-growing broilers (63.0-80.0%), male layer hybrids (76.9-97.6%) and dual-purpose cockerels (69.0-89.6%). Resistance to ampicillin (11.1%; 77/696), ciprofloxacin (9.9%; 69/696), tetracycline (9.5%; 66/696), and nalidixic acid (8.3%; 58/696) was overall most prevalent in resistant commensal isolates regardless of sampling time point and fattening type. Whole genome sequencing revealed a diverse population among resistant commensal *E. coli*, with most resistant strains belonging to ST10 or ST155. Clonal dissemination of resistant strains was shown both within flocks or between subsequent flocks of a farm and between different farms. The providing hatchery was shown to have an influence (*p* < 0.001) on the recovery of resistant isolates. Only 27.5% of all flocks were positive for ESBL-/AmpC-producing *E. coli* for at least one sampling time point, with none of the flocks being positive throughout the whole fattening period and a high diversity of sequence types. *bla*_CTX-M-1_ (29.2%, 7/24) was the most prevalent ESBL gene identified. This study is the first to describe antimicrobial resistance in different organic meat chicken fattening types in Germany in a longitudinal approach.

## Introduction

Antimicrobial resistance (AMR) remains a global One Health challenge: Infections with antimicrobial resistant bacteria lead to limited treatment options, increased mortality and high socioeconomic burden (Maslikowska et al., 2016; Ho et al., 2025). The widespread use of antimicrobials has been a key driver for the development of AMR by exerting selective pressure on the bacterial population and a collaborative approach across human, animal and environmental sectors is needed to combat this development (Prestinaci et al., 2015; Pokharel et al., 2020).

As an intestinal commensal of vertebrates, *Escherichia coli* is used as an indicator organism for monitoring the emergence and prevalence of AMR across different livestock production systems within national surveillance programs (Kaesbohrer et al., 2012). The pathogenic potential of *E. coli* varies by so called pathovars (Croxen and Finlay, 2010). In the veterinary sector, avian pathogenic *E. coli* (APEC) causing colibacillosis are of clinical and economical relevance (Mageiros et al., 2021). The systemic treatment of clinical *E. coli* infections or general use of antibiotics leads to selective pressure on the commensal bacterial population, promoting survival of strains with chromosomal point mutations or strains that have acquired antimicrobial resistance genes through mobile genetic elements, like plasmids, transposons and integrons (Prestinaci et al., 2015). Resistant *E. coli* or mobile genetic elements can further disseminate from humans or livestock into the environment and wildlife through wastewater or manure used to fertilize crops (Zhang et al., 2019; Kumar et al., 2024; Tuts et al., 2025).

The development of reservoirs of resistant bacteria in livestock species and their subsequent transfer through the food chain is of particular concern, as many antimicrobial classes are used in both human and veterinary medicine. Isolates of *E. coli* from cecal content of chicken at slaughter and chicken meat show high rates of AMR and multidrug-resistance (MDR) compared to other livestock species (European Food Safety Authority (EFSA) and European Centre for Disease Prevention and Control (ECDC), 2025), likely linked to the widespread use of different antibiotic classes in broiler production (Roth et al., 2019). While the use of antibiotics as a growth promotor has been banned in the European Union since 2006 and certain antibiotics have been reserved for human treatment only recently (European Commission, 2022), antibiotic treatment and metaphylaxis is otherwise not prohibited in conventional broiler husbandry. The high prevalence of *E. coli* producing either extended-spectrum beta-lactamases (ESBL) or AmpC beta-lactamases, which confer resistance to third and fourth generation cephalosporins, is of particular relevance for public health. ESBL-/AmpC-producing *E. coli* have been shown to be disseminated along all levels of broiler production, including breeder and fattening flocks, hatcheries and day-old chicks (Dierikx et al., 2013; Agersø et al., 2014; Nilsson et al., 2014; Projahn et al., 2017, 2018). The persistence and spread of antimicrobial resistant and ESBL-/AmpC-producing *E. coli* in broiler production pose the risk of transmission to workers on broiler farms, into the slaughterhouse and finally to consumers, who might consume contaminated retail meat (van den Bogaard et al., 2001; Tippelskirch et al., 2018).

In the European Union, organic production is regulated by Regulation (EU) No. 2018/848 and is characterized by use of slow-growing broiler breeds, defined as broilers with a daily growth rate of a maximum of 80% of the conventional high efficiency breeds (Länderarbeitsgemeinschaft Ökologischer Landbau (LÖK), 2009), or a higher slaughter age of at least 81 days. Additionally, free-range farming is mandatory for at least a third of the chicken's lifespan, flock sizes are smaller with lower stocking densities (of max. 21 kg per m^2^), and feed has to be produced organically and locally. Antimicrobial use is strictly regulated and only permitted once during the fattening period (European Parliament, 2018). Organic meat chicken production thus represents a diverse system with higher exposure to the environment than in conventional production, different chicken hybrid lineages and less antimicrobial selection pressure. Although the number of organically raised meat chicken has steadily increased over the last decade, the organic meat chicken sector still represents a small share of overall farms and number of chickens slaughtered within the European Union (European Commission, 2023; Eurostat, 2025). Consequently, their contribution to annual monitoring results is negligible and differences in AMR prevalence between conventional and organic meat chicken are not regularly evaluated. Comparative analysis from German and Austrian national AMR surveillance demonstrated, that AMR *E. coli* could still be retrieved from organic flocks on farm and from caecal content collected in slaughterhouses, but that the odds of retrieving susceptible isolates were significantly higher compared to conventional flocks (Much et al., 2019; Grobbel et al., 2022;). Similar differences were observed in Italy, were ESBL-/AmpC-producing *E. coli* could be retrieved from both conventional and organic flocks at slaughter in Italy, but broilers from organic flocks harbored less antimicrobial resistant *E. coli* (Musa et al., 2020; Pesciaroli et al., 2020). Another Italian study demonstrated, that the load of ESBL-/AmpC-producing *E. coli* in the gut was significantly lower in organic flocks (Tofani et al., 2022). However, a similar prevalence of ESBL-producing *E. coli* in organic broiler farms and corresponding workers on farm compared to conventional farms was reported in the Netherlands (Huijbers et al., 2015). In the Netherlands, studies on transmission dynamics and carriage of EBSL-producing *E. coli* in two successive organic broiler flocks showed that similar to conventional broiler, day-old chicks were already carriers (Huijbers et al., 2016; van Hoek et al., 2018). Similarly, we demonstrated repeated introduction of MDR *E. coli* with day-old chicks into a German organic broiler farm (Korves et al., 2025).

The majority of studies regarding the differences in antimicrobial resistant, multidrug-resistant and ESBL-/AmpC-producing *E. coli* between the organic and conventional sector focus on chicken meat at retail (Miranda et al., 2008; Cohen Stuart et al., 2012; Kola et al., 2012; Millman et al., 2013; Mollenkopf et al., 2014;Davis et al., 2018; Kim et al., 2020; Uyanik et al., 2021; Vieira et al., 2022), where the influence of strains introduced due to cross-contamination along the slaughter line must be taken into consideration. Consequently, there is still much to discover about the origin and transmission dynamics of AMR *E. coli* in organic meat chicken farms, considering both the influence of providing hatchery, use of different chicken lineages and outdoor access. The authors are not aware of studies investigating AMR prevalence in male layer hybrids or dual-purpose cockerels fattened as organic meat chickens, nor of studies targeting organic breeder flocks or hatcheries.

We designed a longitudinal study comparing three different organic chicken types used for meat production: slow-growing broilers, male layer hybrids and dual-purpose cockerels. To account for different chick origins, chicken ages and management differences, several farms per chicken fattening type were acquired for the project and sampled over four consecutive fattening flocks each. The study aimed to:

1) Assess changes in the antimicrobial resistance of commensal *E. coli* and prevalence of ESBL-/AmpC-producing *E. coli* throughout the fattening period
2) Characterize resistant strains regarding phylogenetic relation and resistance determinants
3) Evaluate different fattening types, sampling time point and supplying hatcheries as risk factors for introduction of resistant isolates

## Material and methods

### Organic farms and fattening types

Fourteen certified organic meat chicken farms in Germany were acquired for this study as part of the ProBioHuhn project. The majority of farms was located in Western and Central Germany (7 in Hesse, 5 in Lower Saxony, 2 in other states). Of each farm, one fattening type was included in the study: slow-growing broilers (*n* = 5), male layer hybrids (*n* = 5) or dual-purpose cockerels (*n* = 4). Each farm within a category used the same chicken hybrids: slow-growing broilers were represented by ISA JA 757 or ISA JA 87 and ISA CY 57, male layer hybrids by Lohmann Brown-Plus and dual-purpose cockerels by “Coffee & Cream” (Ökologische Tierzucht gGmbH, Augsburg, Germany). Four consecutive flocks were sampled per farm, except farms 5 and 14. Farm 5 stopped the fattening of male layer hybrids after two flocks during the project, therefore one flock of Dekalb White chickens (farm 14) was included as an additional male layer hybrid flock.

Farm 8 did not rear dual-purpose cockerels themselves but specialized in finishing chickens bought from farm 6 and 9 in a mobile house. For flock 1 on farm 8, chickens from flock 2 on farm 6 were rehoused between sampling time point (S) 3 and S4. Flock 2 on farm 8 was provided by farm 6 from a flock, that was not part of an independent sampling flock in the project. For flock 3 on farm 8, chicken from flock 4 of farm 9 were rehoused between S3 and S4. Flock 4 on farm 8 was provided by farm 9 from a flock that was not part of an independent sampling flock in the project.

### Hatcheries

Flocks originated from six different hatcheries, with the majority of flocks being provided by hatchery H2 and H4. All hatcheries were certified organic. With the exception of H6 (Netherlands), all hatcheries were located in Germany (Supplements 2).

### Sampling

51 flocks were sampled from February 2023 to January 2025 at five sampling time points (S) per flock. Since age of slaughter and hence the length of the fattening period differed greatly between different fattening types and farms (Table 1), sampling time points represented important time periods during rearing and fattening rather than fixed days of life for comparison between flocks. Briefly, meconium covered inlay papers or litter from transport boxes of chicks were sampled upon arrival on farm (S1). For each subsequent sampling time point, fecal and environmental samples consisted of one pair of boot swabs (10001911, Romer Labs Division Holding GmbH, Getzersdorf, Austria) taken from the indoor housing area of the flock monitored. The rearing house was sampled within the second week (10-14 days of age) on farm (S2) and after approximately four weeks (30±4 days) before outdoor access was granted (S3). The housing compartments of flocks were sampled again two weeks after first outdoor access to pasture (S4) and upon reaching age of slaughter (S5), with sampling taking place one to two weeks before slaughter or one to two weeks after the first thinning, if the flock was slaughtered in multiple batches. All samples were immediately placed into padded envelopes containing a cooling pack and sent to the National Reference Laboratory for Antimicrobial Resistance by 24 h express delivery. Samples were processed within 96 hours of arrival.

**Table 1:** Farms included into the study with fattening type and breed used, number of sampled flocks, number of birds per flock at sampling time point (S) 1 (minimum – maximum) and mean age of birds at S1 to 5.

Farm ID	Fattening type	Chicken breed	No. of flocks	Birds per flock	Age at S1	Age at S2	Age at S3	Age at S4	Age at S5
1	Slow-growing broilers	ISA JA 757	4	102-204	1d	11d	31d	67d	89d
2	Slow-growing broilers	ISA JA 757, ISA JA 87 CY, ISA CY57	4	4800	1d	13d	29d	48d	63d
3	Slow-growing broilers	ISA JA 757	4	350-459	1d	13d	33d	58d	84d
4	Dual-purpose cockerels	Coffee & Cream	4	660-700	1d	13d	31d	76d	121d
5	Male layers	Lohmann Brown +	2	500-1500	1d	14d	33d	66d	102d
6	Dual-purpose cockerels	Coffee & Cream	4	682-1048	1d	12d	33d	78d	111d
7	Slow-growing broilers	ISA CY57	4	1020-1184	1d	11d	30d	77d	/a
8	Dual-purpose cockerels	Coffee & Cream	4	70-101	1d^b^	13d^b^	31d^b^	71d	105d
9	Dual-purpose cockerels	Coffee & Cream	4	1000-4390	1d	13d	30d	83d	132d
10	Slow-growing broilers	ISA JA 757, ISA CY57	4	2200	1d	10d	31d	65d	80d
11	Male layers	Lohmann Brown +	4	4800-6750	1d	13d	33d	88d^c^	111d^c^
12	Male layers	Lohmann Brown +	4	3500-3550	1d	13d	32d	56d	81d
13	Male layers	Lohmann Brown +	4	1263-2326	1d	15d^d^	31d	81d	107d
14	Male layers	Dekalb White	1	480	3d	9d^e^	31d	63d	100d

a S5 did not take place at farm 7 due to only providing first outdoor access closely before first slaughter.

b chickens were reared on farms 6 and 9 for S1-3.

c S4 and S5 did not take place for the last two flocks.

d one flock was sampled after 17 days for S2 due to scheduling issues.

e flock was sampled after 9 days for S2 due to scheduling issues S1: chicks upon delivery; S2: first two weeks of life; S3: first four weeks of life; S4: two weeks after first outdoor access; S5: age of slaughter.

### Antimicrobial treatment of flocks

During the study, three flocks received antimicrobial treatment:

On farm 10, flock 2 was treated with toltrazuril (0.2 ml per bird and day for two days) and tylosin (0.01 g per bird and day for four days) after a diagnosis of coccidiosis and necrotizing enteritis. The subsequent flock 3 was again treated with the same dosage of tylosin for five days after a diagnosis of chronic airway infection. The treatment of both flocks occurred between S3 and S4.

On farm 2, flock 4 was treated with amprolium (0.5ml/kg) for coccidiosis and amoxicillin (15mg/kg) after a positive *Clostridium* spp. result according to the contracted veterinarian, but no further details regarding the duration of the medication could be received. The treatment occurred between S2 and S3.

### Isolation, identification and storage of isolates

Isolation of *E. coli* isolates from meconium samples and boot swabs on MacConkey agar (MCA) and MacConkey agar + 1mg/L Cefotaxime (MCA+CTX), species confirmation through MALDI ToF (Maldi BioTyper microflex LT/SH, Bruker Corporation, Billerica, MA) and storage of isolates was performed as described previously (Korves et al., 2025). Briefly, the meconium samples or pair of boot swabs from each sampling time point were pooled and enriched aerobically at 37°C for 18–22 hours in peptone water. The enrichment broth was then cultivated on MCA and MCA+CTX at 44°C for 18-22 hours. Five colonies with typical *E. coli* morphology were chosen from each plate for subcultivation on Columbia agar supplemented with 5% sheep blood for MALDI ToF identification. Three *E. coli* per plate and sample with a MALDI ToF score ≥2.3 and varying colony morphology (if present) were isolated for antimicrobial susceptibility testing.

### Antimicrobial susceptibility testing

Antimicrobial susceptibility testing was conducted through broth microdilution on commercial EUVSEC3 plates (Sensititre™, Trek Diagnostic Systems Ltd., East Grinstead, United Kingdom) for all isolates according to the guidelines of the Clinical and Laboratory Standards Institute (CLSI) (CLSI, 2018). The plates contained the harmonized testing panel of 15 antibiotics from 12 antimicrobial classes for European AMR surveillance: ampicillin (1-32 mg/L), amikacin (4-128 mg/L), azithromycin (2-64 mg/L), cefotaxime (0.25-4 mg/L), ceftazidime (0.25-8 mg/L), ciprofloxacin (0.015-8 mg/L), colistin (1-16 mg/L), gentamicin (0.5-16 mg/L), meropenem (0.03-16 mg/L), nalidixic acid (4-64 mg/L), sulfamethoxazole (8-512 mg/L), tetracycline (2-32 mg/L), tigecycline (0.25-8 mg/L) and trimethoprim (0.25-16 mg/L) as described in Commission Implementing Decision (EU) 2020/1729 (European Commission, 2020). Minimum inhibitory concentrations were evaluated using epidemiological cut-off values (ECOFF) as described in Commission Implementing Decision (EU) 2020/1729 and specifications for data reporting from the European Food Safety Authority (EFSA) (EFSA, 2021). Isolates with no phenotypical resistance according to the testing panel were defined as “fully susceptible”. “Multidrug resistance” was defined as resistance to substances from three or more antimicrobial classes (Magiorakos et al., 2012).

### DNA extraction and whole genome sequencing

All suspected ESBL-/AmpC- producing *E. coli* obtained from MCA+CTX (*n* = 51) were submitted to Whole Genome Sequencing (WGS). For otherwise resistant *E. coli* isolates from MCA, one isolate per unique resistogramme was chosen per sample, resulting in another 127 isolates.

Isolates were cultured in LB broth overnight (37°C, aerobic). The next day, 1 mL of cultured broth was centrifuged down at 13.300 rpm for five minutes to obtain cell pellets. Cell pellets were either temporally stored at −20°C or immediately processed using the PureLink^TM^ Genomic DNA Mini Kit (Invitrogen, Life Technologies Corp, Carlsbad, CA) for DNA extraction according to manufacturer’s instructions.

The sequencing libraries were prepared using the Illumina DNA Flex Library Preparation Kit (Illumina Inc., Sandiego, CA), followed by a paired-end sequencing run (2 × 150 bp) on a NextSeq500 or MiSeq device (Illumina Inc., Sandiego, CA) according to manufacturer’s protocols. Trimming, *de novo* assembly of raw short reads and quality control was performed through the AQUAMIS-pipeline (versions 1.3.8, 1.4.1 and 1.4.2) (https://gitlab.com/bfr_bioinformatics/AQUAMIS/) (Deneke et al., 2021a).

### Confirmation of ESBL-/AmpC-producing *E. coli* and bioinformatic analysis

Presence of ESBL-/AmpC-genes or chromosomal point mutations of AmpC promotor region was verified in CTX-resistant isolates through bioinformatical analysis of short read sequencing data described below.

Bioinformatic analyses were carried out using genome assemblies in fasta-format, unless specified otherwise. The bakcharak pipeline version 3.0.4 (https://gitlab.com/bfr_bioinformatics/bakcharak) was used to analyze genotype based on phylogroup and MLST, and to identify antimicrobial resistance genes. For nine isolates, no MLST could be assigned through bakcharak. These assemblies were reanalyzed in Enterobase (https://enterobase.warwick.ac.uk) and were identified as ST17368, ST17739, ST17740, ST17741, ST17742, ST17744 (*n* = 2), ST17745 and ST17746 (Zhou et al., 2020). All isolates were additionally analyzed with standard settings (threshold for ID 90%, minimum length 60%) in ResFinder version 2.4.0 (https://genepi.dk/resfinder) of the Center for Genomic Epidemiology to account for differences between the AMRFinder and ResFinder database (Camacho et al., 2009; Bortolaia et al., 2020). In case of discrepancies, the closest result based on gene coverage and identity was chosen. The phylogenetic structure of the isolates was investigated using the chewiesnake pipeline (https://gitlab.com/bfr_bioinformatics/chewieSnake) for cgMLST allele calling based on 2514 loci (Deneke et al., 2021b). Isolates with a cgMLST allele difference (AD) ≤10 were examined through snippysnake (https://gitlab.com/bfr_bioinformatics/snippySnake) to identify single nucleotide polymorphisms (SNP) (Deneke, 2019; Lüth et al., 2021). SNP-based analysis was conducted with trimmed fastq-files using the isolate first detected within a putative cluster as a reference (Jamin et al., 2021). Clonal clusters were defined using the following thresholds: cgMLST AD ≤10, SNP ≤20 (Pightling et al., 2018) and shared reference genome ≥95%. All assemblies were uploaded to the Genebank of the National Center for Biotechnology Information (NCBI) under bioproject PRJNA1290230.

### Visualization

Graphs were constructed using GraphPad Prism (version 10.1.2 (324)). Fig.s of phylogenetic trees were visualized using iTol v7 (Letunic and Bork, 2024).

### Statistical analyses

A Generalized Linear Mixed Modell (GLMM) was used for statistical analyses in IBM® SPSS® Statistics 31 using a binominal distribution with a logit link to examine the effects of fattening type, sampling time point (indicating age group as well as outdoor access), supplying hatchery and antimicrobial treatment on the recovery of resistant *E. coli* from unselective. MCA. Antimicrobial resistance of each *E. coli* isolate was coded as a binary outcome variable (0 = susceptible; 1 = one or more resistances). Due to low number of resistant isolates compared to total number of isolates retrieved through unselective cultivation on MCA (149/696), only categorical variables deemed most important to compare groups based on sampling scheme and analysis of clonal clusters were included into the model as fixed effects. For sampling time point, S5 (age of slaughter) was used as a reference category. For fattening type, male layer hybrids were used as a reference category. For the supplying hatchery, hatchery H2 supplying chicks for most of the slow-growing broiler flocks was used as a reference category. The farm*flock interaction was included as a random intercept. A p-value <0.05 was considered statistically significant.

The model fitness was assessed using corrected Akaike information criterion (AICc), Bayes information criterion (BIC) and Pseudo-R^2^ measures (Nakagawa and Schielzeth, 2013).

Assessing the same fixed and random effects for MDR of *E. coli* isolates (38/696) did not result in stable models and was therefore not considered further.

## Results

### Samples and isolation of *E. coli*

Between February 2023 and January 2025, 236 samples arrived at the National Reference Laboratory for antimicrobial resistance for culturing (Table 2). All boot swabs (*n* = 189) showed *E. coli* growth on MCA, but only 91.5% (43/47) of meconium-covered inlay papers. 696 isolates were obtained from non-selective MCA and another 51 suspected ESBL-/AmpC-producing *E. coli* from selective MCA+CTX.

**Table 2:** Number of samples examined and *E. coli* isolated for each flock.

	**Meconium samples**			**Boot swabs**				
**Farm**	**Samples**	**Positive for commensal *E. coli***	**Positive for ESBL/AmpC *E. coli***	**Samples**	**Positive for commensal *E. coli***	**Positive for ESBL/AmpC *E. coli***	**Total number of commensal *E. coli***	**Total number of ESBL/AmpC *E. coli***
1^a^	4	3	0	16	16	0	57	0
2^I^^,^^II^	3	3	0	14	14	2	51	6
3	4	4	0	16	16	0	60	0
4^b^	4	2	0	16	16	6	54	13
5III	1	1	0	8	8	0	27	0
6^IV^	4	4	0	15	15	0	57	0
7^V^	4	4	0	12	12	1	48	3
8^VI^^,^^c^	2	1	0	12	12	3	39	7
9	4	4	0	16	16	1	60	3
10	4	4	0	16	16	0	60	0
11VII	4	4	0	12	12	2	48	4
12	4	4	0	16	16	2	60	5
13	4	4	0	16	16	2	60	5
14	1	1	1	4	4	1	15	5
								
**Total**	47	43	1	189	189	20	696	51

I day-old chicks of flock 3 were transported without inlay-papers or litter, no sample was taken.

II sampling time point 4 of flock 3 and sampling time point 5 of flock 4 were missed.

III only two flocks were sampled, first meconium sampling was missed due to early delivery of chicks.

IV sampling time point 4 of flock 3 was missed.

V sampling time point 5 was missed in all flocks due to early slaughtering of birds.

VI chicks from flocks of farms 6 and 9 were only fattened on farm for S4 and S5; flock 1 came from project flock 2 farm 6, flock 2 was reared separately on farm 6, flock 3 came from project flock 4 farm 9, flock 4 was reared separately on farm 9.

VII sampling time points 4 and 5 were missed in flocks 3 and 4.

a meconium sample of flock 4 negative for *Escherichia-like* growth.

b meconium sample of flock 3 negative for *Escherichia-like* growth, meconium sample of flock 4 showed only growth of *E. marmotae*.

c meconium sample of flock 2 negative for *Escherichia*-like growth.

### Antimicrobial susceptibility of indicator commensal *E. coli* between different fattening types and over time

All 696 isolates obtained from non-selective MCA were submitted to phenotypic antimicrobial susceptibility testing. Overall, 78.6% (547/696) of isolates were fully susceptible to all antimicrobial substances tested. The resistant isolates predominantly showed resistance to one antimicrobial class (11.9%, 83/696), while resistance to two antimicrobial classes (4.0%, 28/696) or multi-resistance (5.5%, 38/696) was less prevalent.

**Fig. 1:**
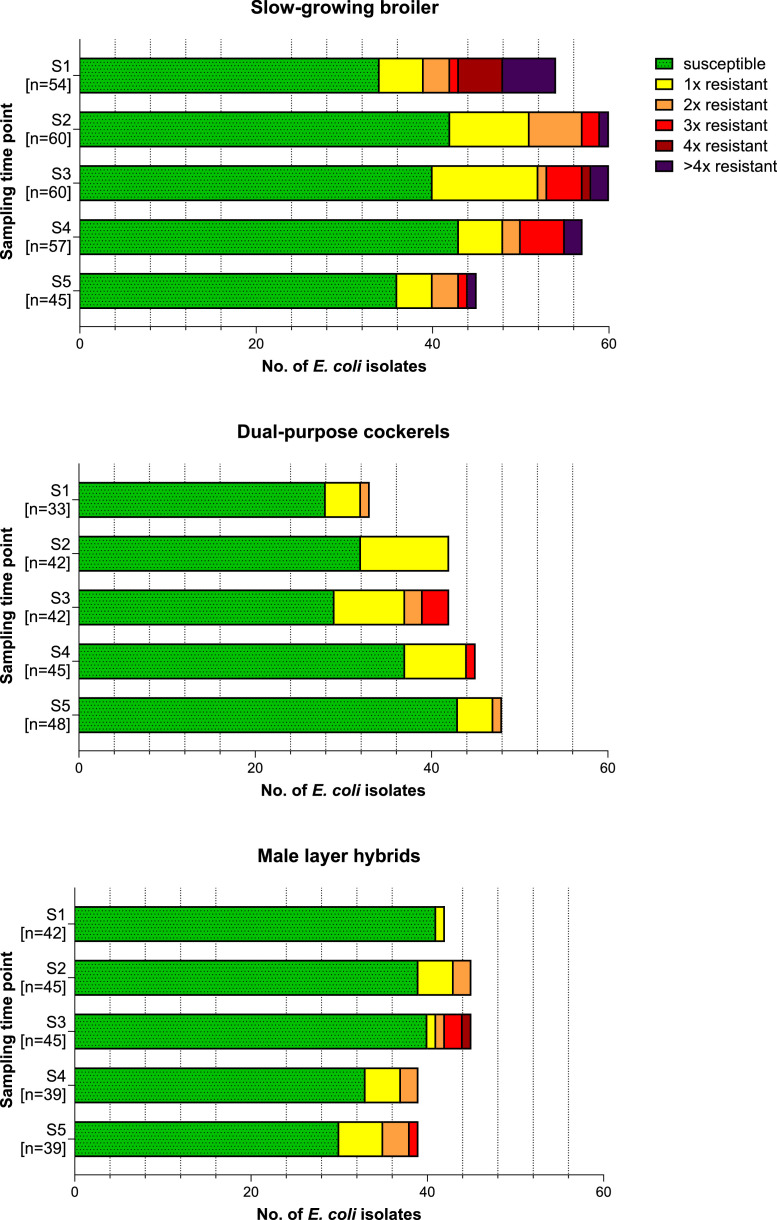
Number (n) of resistant *E. coli* per fattening type and sampling time point S1-S5. Three isolates from MCA per sample (resulting in 696 isolates) were chosen for analysis of resistance to 15 antimicrobial substances from 12 antimicrobial classes determined via broth microdilution. The number of resistances to different antimicrobial classes per isolate is depicted as a stacked bar chart representing the number of the overall isolates from a respective sampling time point S1-S5. Isolates not phenotypically resistant to antimicrobial classes from the testing panel are categorized as susceptible and depicted in green.

This distribution was reflected in all three fattening types throughout the fattening period, with fully susceptible isolates accounting for the largest share at each sampling time point (Fig. 1). Day-old chicks (S1) of male layer hybrids exhibited the highest share of fully susceptible isolates with 97.6% (41/42). The rate of full susceptibility decreased over the fattening period in male layer hybrids. In contrast, isolates from day-old chicks (S1) of slow-growing broiler exhibited the lowest share of fully susceptible isolates with 63.0% (34/54), but the number of susceptible isolates increased over the course of fattening.

Correspondingly, multi-resistant isolates were primarily associated with slow-growing broilers at all sampling time points, with day-old chicks (S1) exhibiting the highest share of all examined groups (22.2%, 12/54). Especially isolates carrying resistance to four or more different antimicrobial classes were almost exclusively detected in slow-growing broilers.

**Fig. 2:**
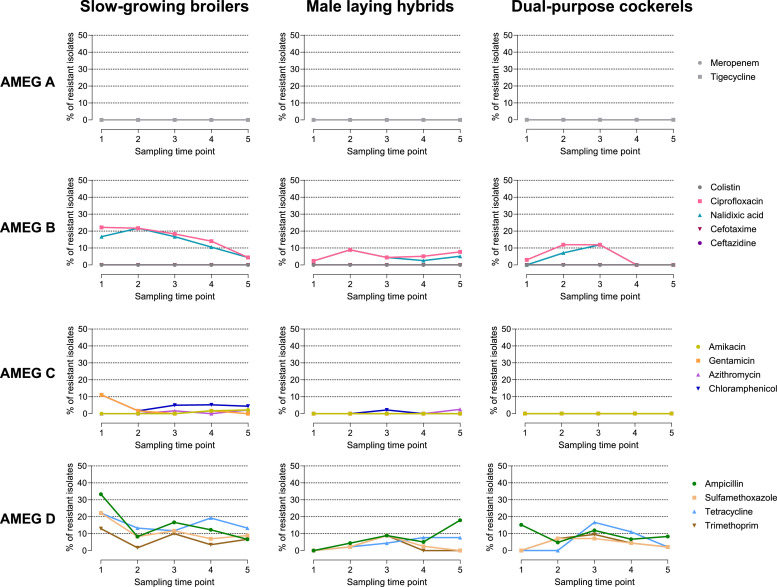
Share (%) of all 696 *E. coli* isolates phenotypically resistant to different antimicrobial substances sorted by AMEG-categorization and fattening type over the fattening period.

Resistance to antibiotics in AMEG categories B and D was especially pronounced in slow-growing broilers (Fig. 2), with the share of resistant isolates against ampicillin (AMP), sulfamethoxazole (SMX), tetracycline (TET), trimethoprim (TMP), ciprofloxacin (CIP) and nalidixic acid (NAL) steadily decreasing over the course of fattening. For instance, 33.3% (18/54) of isolates from day-old chicks (S1) of slow-growing broilers were resistant to AMP, whereas only 6.7% (3/45) of isolates from slow-growing broiler at age of slaughter (S5) exhibited resistance to AMP. As for general distribution of resistant isolates, the opposite trend could be observed in male layer hybrids, where resistance to AMP increased from none in day-old chicks (S1) to 17.9% (7/39) at age of slaughter (S5).

### Resistance determinants and dissemination of resistant commensal *E. coli*

**Fig. 3:**
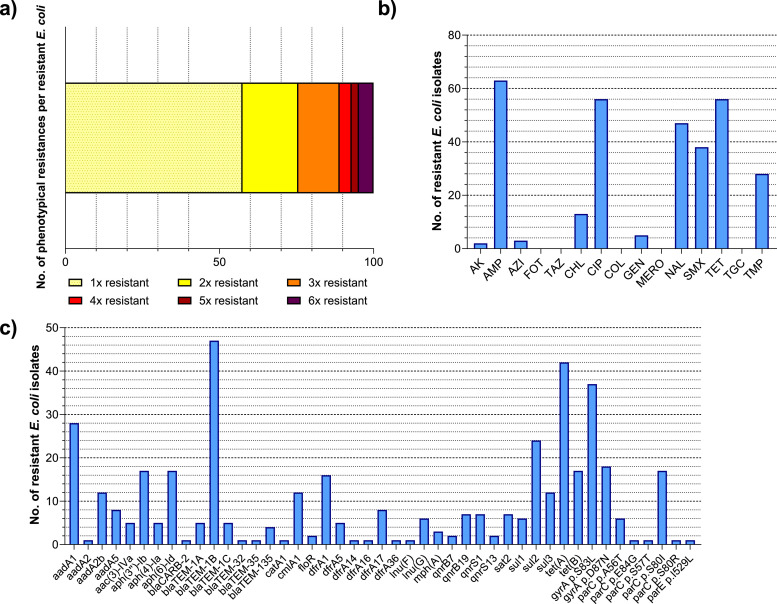
Antimicrobial resistance and antimicrobial resistant genes of 127 ESBL-/AmpC-producing *E. coli*. One isolate per resistogram and sampling time point (*n* = 127 isolates) was chosen for analysis of a) resistance to 15 antimicrobial substances determined via broth microdilution, b) number of resistances to different antimicrobial classes per isolate and c) presence of antimicrobial resistance genes or chromosomal point mutations causing resistance determined through whole genome sequencing. *AK: amikacin, AMP: ampicillin, AZI: azithromycin, FOT: cefotaxime, TAZ: ceftazidime, CHL: chloramphenicol, CIP: ciprofloxacin, COL: colistin, GEN: gentamicin, MERO: meropenem, NAL: nalidixic acid, SMX: sulfamethoxazole, TET: tetracycline, TGC: tigecycline, TMP: trimethoprim.*

**Fig. 4:**
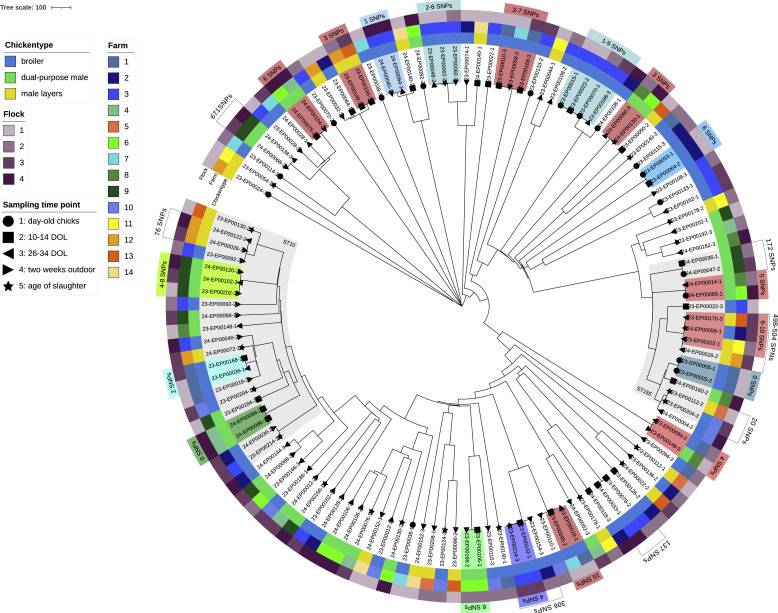
Genetic diversity and clusters of 127 resistant commensal *E. coli*. The tree was calculated based on cgMLST with chewiesnake and constructed in iTol v7. Clonal clusters were assumed for *a* ≤ 10 allele difference. Clonality of clustering isolates was further investigated through CSI phylogeny using ≤20 SNPs and ≥95% shared reference genome as thresholds. Clusters of clonal isolates within the same farm are highlighted in green or blue depending on the fattening type, clusters of clonal isolates across different farms are highlighted in red. Putative clusters isolates with *a* ≤ 10 cgMLST allele difference and higher number of SNPs are highlighted with a bracket.

Based on phenotypical antimicrobial susceptibility, one *E. coli* isolate per unique resistogram and sampling time point was chosen for WGS and analysis of antimicrobial resistance genes, phylogenetic relationships and clusters of clonal isolates, resulting in 127 isolates (Fig. 3, Fig. 4).

In line with the results for all tested commensal *E. coli*, the share of isolates with resistance to one antimicrobial class was highest among resistant *E. coli* with 57.5% (73/127) (Fig. 3a).

Resistance to ampicillin was most prevalent with 49.6% (63/127), followed by ciprofloxacin and tetracycline (both 44.1%, 56/127) and nalidixic acid (37.0%, 47/127) (Fig. 3b). Accordingly, *bla*_TEM-1B_ was the most common resistance gene detected (Fig. 3c). Resistance to ciprofloxacin (CIP) and nalidixic acid (NAL) was mostly associated with chromosomal point mutation *gyrA* p.S83L (29.1%, 37/127), while different *qnr*-genes were only present in 14.2% (18/127) of isolates. In two cases, two isolates picked from the same sample showed different resistogrammes despite harbouring the same genetic resistance determinants, including *qnrS13* resp. *qnrB19*, as the minimum inhibitory concentrations (MIC) were close to the cut-off value of 16 mg/L. Chromosomal point mutation *parE* p.I529L was identified in one isolate not phenotypically resistant against CIP (see Supplements 1). Among tetracycline resistance genes, *tet(A)* was the most prevalent with 33.1% (42/127), followed by *tet(B*) (13.4%, 17/127). Resistance to folate pathway inhibitors sulfamethoxazole and trimethoprim was relatively common with 29.9% (38/127) and 22.0% (28/127), mostly associated with antimicrobial resistance genes *sul2* and *dfr1*. Phenotypic resistance to chloramphenicol (10.2%, 13/127), azithromycin (2.4%, 3/127) as well as aminoglycosides gentamicin (3.9%, 5/127) and amikacin (1.6%, 2/127) was rare among resistant isolates. However, resistance genes against different aminoglycosides were commonly detected through WGS, with the most common being *aadA1* (22.0%, 28/127), *aph(3″)-Ib* and *aph(6)-Id* (both 13.4%, 17/127).

No resistance to cefotaxime, ceftazidime, colistin, meropenem or tigecycline was detected in commensal *E. coli* isolates picked from MCA.

The resistant *E. coli* population exhibited a diverse genetic background with isolates belonging to eight different phylogroups and 58 different MLST. Phylogroup A was by far the most common phylogroup with 53.5% (68/127) of isolates, followed by B1 (26.0%, 33/127), D and G (5.5% each, 7/127). Phylogroups B2, C, E and F were represented each with less than 5% of isolates. Correspondingly, ST10 and ST155 were by far the most prevalent MLST overall, making up 16.5% (21/127) and 11.0% (14/127) of isolates, respectively. All other MLST were represented with 0.8% (1/127) to 3.9% (5/127) of isolates (see Supplements 1).

Based on cgMLST allele difference (≤10) and SNP calling (≤20), 18 clonal clusters were identified. Most of the clusters were composed of two isolates (*n* = 13), the largest cluster (h) contained four isolates (Fig. 4, Table 3). All isolates in each cluster belonged to the same respective MLST, with three clusters (a-c) belonging to ST10 and three clusters (f, III, IV) belonging to ST155.

**Table 3:** Clonal clusters identified among 127 resistant *E. coli* isolates through cgMLST allele difference, shared reference genome and SNPs.

**Cluster**	**Isolates**	**SNPs**	**Shared Genome**	**cgMLST AD**	**MLST**	**Source**	**Hatchery**	**Phenotypical resistance**	**Resistance determinants**
a	23-EP00202-3	4	99.18	2	ST10	F9-Fl1-S3	H4	AMP, SMX, TMP	*aph(3′')-Ib; aph(6)-Id; blaTEM-1B; dfrA5; sul2*
	24-EP00102-3								
	24-EP00102-3	6		2		F9-Fl3-S4		AMP, SMX, TET, TMP	*aph(3′')-Ib; aph(6)-Id; blaTEM-1B; dfrA5; sul2; tet(A)*
	24-EP00120-2								
	24-EP00120-2	8		4		F9-Fl4-S3		AMP, SMX, TET, TMP	*aph(3′')-Ib; aph(6)-Id; blaTEM-1B; dfrA5; sul2; tet(A)*
	23-EP00202-3								

b	23-EP00036-1	2	99.16	1	ST10	F1-Fl1-S4	H2	TET	*tet(A)*
	23-EP00168-1					F1-Fl3-S2			

c	24-EP00086-1	0	99.61	0	ST10	F4-Fl4-S2	H3	CIP, NAL	*qnrB19*
	24-EP00086-2							CIP	

d	23-EP00106-1	6	98.87	2	ST3856	F6-Fl2-S2	H3	SMX, TMP	*aadA5; dfrA17; sul2*
	23-EP00166-2					F6-Fl2-S4			

e	23-EP00142-1	4	99.04	1	ST13322	F3-Fl3-S2	H5	CIP, NAL, TET	*tet(B); gyrA* p.S83L*; gyrA* p.D87N*; parC* p.A56T*; parC* p.S80I
	23-EP00214-3					F3-Fl3-S5			

f	23-EP00005-1	0	99.14	0	ST155	F1-Fl1-S1	H2	AMP, CIP, TET, TMP	*aadA1; blaTEM-1B; dfrA1; qnrS13; tet(A)*
	23-EP00005-2							AMP, CIP, NAL, TET, TMP	

g	23-EP00053-1	6	99.20	3	ST442	F2-Fl1-S1	H6	AMP, SMX, TMP	*aadA1; blaTEM-1B; dfrA1; sat2; sul2*
	23-EP00064-2					F2-Fl1-S2			

h	23-EP00015-1	1	99.36	1	ST162	F3-Fl1-S1	H5	AMP, CHL, CIP, GEN, NAL, SMX, TET	*aadA2b; cmlA1; aadA1; sul3; aac(3)-IVa; aph(4)-Ia; tet(A); blaTEM-1B; gyrA* p.S83L*; gyrA* p.D87N*; parC* p.S80I
	23-EP00022-2					F3-Fl1-S2			
	23-EP00015-1	4		1					
	23-EP00070-1					F3-Fl2-S1			
	23-EP00015-1	8		3					
	23-EP00188-3					F3-Fl3-S4			
	23-EP00022-2	5		2					
	23-EP00070-1								
	23-EP00022-2	9		3					
	23-EP00188-3								
	23-EP00070-1	6		2					
	23-EP00188-3								

i	23-EP00048-2	2	98.76	0	ST57	F3-Fl1-S4	H5	AK, AMP, CHL, CIP, NAL, SMX, TET, TMP	*aadA1; aadA1; aadA2b; aph(3′')-Ib; aph(6)-Id; blaTEM-1A; cmlA1; dfrA1; sat2; sul3; tet(B); gyrA* p.S83L*; gyrA* p.D87N*; parC* p.S80I
	23-EP00062-2								
	23-EP00062-2	6		2		F3-Fl1-S5		AK, AMP, CHL, CIP, NAL, SMX, TET, TMP	*aadA1; aadA1; aadA2b; aph(3′')-Ib; aph(6)-Id; blaTEM-1A; cmlA1; dfrA1; sat2; sul3; tet(B); gyrA* p.S83L*; gyrA* p.D87N*; parC* p.S80I
	23-EP00092-1								
	23-EP00092-1	6		2		F3-Fl2-S3		AMP, CHL, CIP, NAL, SMX, TET, TMP	*aadA1; aadA2b; aph(3′')-Ib; aph(6)-Id; blaTEM-1A; cmlA1; dfrA1; sat2; sul3; tet(B); gyrA* p.S83L*; gyrA* p.D87N*; parC* p.S80I
	23-EP00048-2								

j	24-EP00040-1	1	99.23	0	ST69	F3-Fl4-S3	H2	AMP	*blaTEM-1B*
	24-EP00064-1					F3-Fl4-S4			

I	23-EP00018-1	15	95.55	6	ST8132	F1-Fl1-S3	H2	CIP, NAL, SMX, TET	*sul2; tet(A); tet(B); gyrA* p.S83L*; parC* p.A56T
	23-EP00045-2					F7-Fl1-S2		CIP, NAL, TET	*tet(B); gyrA* p.S83L*; parC* p.A56T

II	23-EP00094-2	4	97.53	0	ST5416	F1-Fl2-S3	H2	TET	*tet(A)*
	23-EP00148-3					F8-Fl1-S4	H3		

III	23-EP00102-1	8	99.31	7	ST155	F11-Fl2-S3	H4	AMP, SMX, TET, TMP	*aadA1; aadA5; aph(3′')-Ib; aph(6)-Id; blaTEM-1B; dfrA1; dfrA17; lnu(G); sul1; sul2; tet(A)*
	23-EP00170-3					F13-Fl3-S3			
	23-EP00102-1	10		6					
	24-EP00056-1					F2-Fl3-S5			
	23-EP00170-3	6		3					
	24-EP00056-1								

IV	24-EP00014-1	5	98.58	2	ST155	F8-Fl2-S3	H3	AMP	*blaTEM-1A*
	24-EP00065-1					F6-Fl4-S1			

V	24-EP00060-3	3	99.53	1	ST1730	F8-Fl2-S4	H3	TET	*aph(3′')-Ib; aph(6)-Id; tet(A)*
	24-EP00130-1					F6-Fl4-S5			

VI	23-EP00010-3	7	95.60	1	ST9721	F1-Fl1-S2	H2	CIP, NAL	*gyrA* p.S83L
	23-EP00058-2								
	23-EP00058-2	7		2		F7-Fl1-S3			
	23-EP00168-3								
	23-EP00168-3	2		1		F1-Fl3-S2			
	23-EP00010-3								

VII	23-EP00149-1	3	96.82	1	ST1485	F12-Fl2-S2	H4	CIP, NAL	*gyrA p.S83L; gyrA* p.D87N*; parC* p.S80I
	23-EP00156-3					F13-Fl3-S2			

VIII	24-EP00075-2	6	98.75	2	ST117	F6-Fl4-S2	H3	CIP, NAL	*gyrA* p.S83L
	24-EP00104-2					F4-Fl4-S3			

F: farm, Fl: flock, S1: chicks upon delivery; S2: first two weeks of life; S3: first four weeks of life; S4: two weeks after first outdoor access; S5: age of slaughter.

Clusters a-j represented clonal isolates disseminated within one flock over several sampling time points (d, e, g, j) or across subsequent flocks of the same farm (a, b, h, i). The two clusters c and f are composed of clonal isolates picked from the same sampling time point due to borderline, and therefore phenotypical inconsistent NAL resistance phenotype associated with the *qnrB19* and *qnrS1* gene, respectively. All other clusters reflect repeated detection of clonal isolates at different sampling time points during the studied time frame. Clusters h and i associated with Farm 3 were found to be dependent on the supplying hatchery H5 and examined in detail in a previous publication (Korves et al., 2025).

Clusters I-VIII represented clonal isolates disseminated between different farms; in two of those cases (II, III) between farms keeping different fattening types. In clusters I, IV, V, VI, VII and VIII, all affected flocks were connected to the same respective hatcheries providing day-old chicks of a certain fattening type to different farms. For cluster III, all affected flocks could be traced back to the same hatchery supplying both male layer hybrid chicks for farms 11 and 13, as well as slow-growing broiler chicks for farm 2. However, only a small number of isolates was sampled from day-old chicks (S1), with most isolates originating from sampling time points S3 to S5. Cluster II contained two ST5416 isolates obtained from farm 1, specialized in raising slow-growing broiler supplied by hatchery H2, and farm 8, specialized in raising dual-purpose cockerels supplied by hatchery H3 and reared in farm 6. Both isolates showed characteristics for clonality with a SNPs difference of 4 and the same resistance profile.

In the majority of all clusters (13/20), isolates shared phenotypic resistance profiles and genetic resistance determinants. For cluster a, a temporal change in the resistance profile through acquisition of mobile genes can be hypothesized: The initial ST10 isolate detected in flock 1 (23-EP00202-3) carried resistance genes *aph(3′')-Ib, aph(6)-Id, bla*_TEM-1B_, *dfrA5* and *sul2*, and demonstrated phenotypic resistance against AMP-SMX-TMP. During the subsequent fattening periods, two clonal ST10 isolates with a 2 to 4 cgMLST allele difference and 6 to 8 SNPs were detected in flock 3 and flock 4. These isolates additionally harbored a *tet(A)* gene and showed phenotypic resistance to TET.

In seven additional cgMLST clusters with an allele difference ≤10, isolates did not meet criteria for clonality due to higher number of SNPs or lower percentage of shared reference genome between the isolates, but still showed some similarity based on resistance profile (see Supplements 2). These putative cases of close genetic relatedness are indicated with grey brackets (Fig. 4).

### Isolation, antimicrobial susceptibility and resistance determinants of ESBL-/AmpC-producing *E. coli*

Selective isolation on MCA+CTX resulted in 8,9% (21/236) positive samples and 51 suspected ESBL-/AmpC-producing *E. coli* isolates (Table 2). Positive samples could be obtained from 9 of the 14 farms. In total, 27.5% (14/51) of sampled flocks were positive on at least one sampling time point, but no flock tested positive on all sampling time points throughout a fattening period. It is interesting to note that, apart from flock 1 on farm 14, no ESBL-/AmpC-producing isolates were detected in chicks (S1). On farm 14, positive results were obtained at both S1 and S2, while subsequent samples were negative for ESBL-/AmpC-producing isolates. The opposite pattern was observed on other farms, where positive results mainly occurred towards the end of the fattening period at S4 (42.9%, 9/21) or S5 (28.6%, 6/21).

**Fig. 5:**
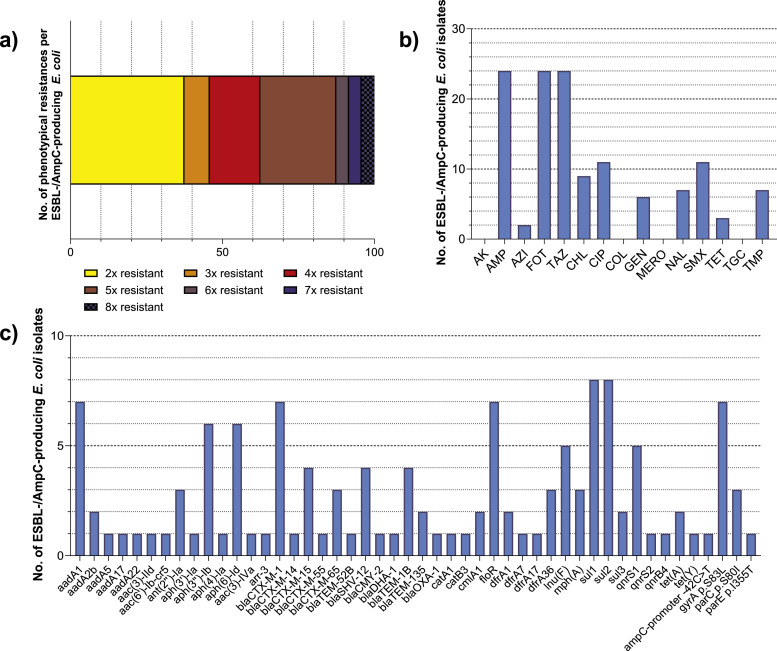
Antimicrobial resistance and antimicrobial resistant genes of 24 ESBL-/AmpC-producing *E. coli*. One isolate per clone, flock and sampling time point (*n* = 24 isolates) was chosen for analysis of a) resistance to 15 antimicrobial substances determined via broth microdilution, b) number of resistances to different antimicrobial classes per isolate and c) presence of antimicrobial resistance genes or chromosomal point mutations causing resistance determined through whole genome sequencing. *AK: amikacin, AMP: ampicillin, AZI: azithromycin, FOT: cefotaxime, TAZ: ceftazidime, CHL: chloramphenicol, CIP: ciprofloxacin, COL: colistin, GEN: gentamicin, MERO: meropenem, NAL: nalidixic acid, SMX: sulfamethoxazole, TET: tetracycline, TGC: tigecycline, TMP: trimethoprim*.

**Fig. 6:**
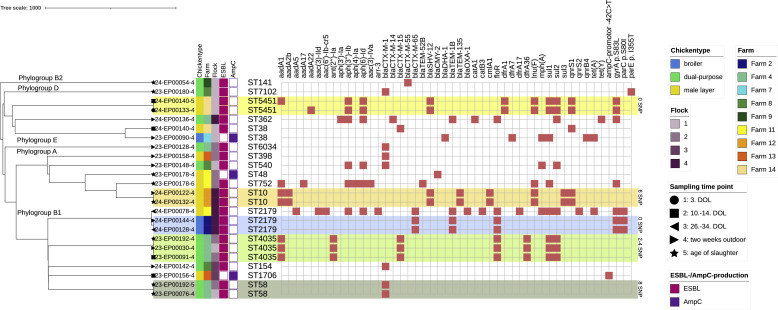
Genetic diversity, resistance genes and chromosomal point mutations of 24 ESBL-/AmpC-producing *E. coli*. The tree was calculated based on cgMLST with chewiesnake and constructed in iTol v7. Clonality of clustering isolates was confirmed through CSI phylogeny using ≤20 SNPs and ≥95% shared reference genome as thresholds. Clusters of clonal isolates are highlighted in color.

All 51 isolates were submitted to phenotypic antimicrobial susceptibility testing and WGS (see Supplements 1). Based on cgMLST and high-resolution SNP-based phylogenetic analyses, one isolate per clone, flock and sampling time point was chosen for final analysis of antimicrobial resistance, antimicrobial resistance genes, phylogenetic relationships and possible clonal spread between flocks and farms, resulting in 24 representative ESBL-/AmpC-producing isolates (Fig. 5, Fig. 6).

ESBL-/AmpC-beta-lactamase production was confirmed in all isolates through presence of corresponding antimicrobial resistance genes and phenotypical resistance to ampicillin, cefotaxime and ceftazidime (Fig. 5). The majority of isolates harbored ESBL (87.5%, 21/24) with the most prevalent ESBL-genes being *bla*_CTX-M-1_ (29.2%, 7/24), *bla*_CTX-M-15_ (16.7%, 4/24) and *bla*_SHV-12_ (16.7%, 4/24). In only three isolates altered or additional AmpC beta-lactamases were found. Here, *bla*_CMY-2_, *bla*_DHA-1_ and chromosomal point mutation of the AmpC-promoter (n-42C>*T*) were represented once each (Fig. 5c).

Overall, most of ESBL-/AmpC-producing isolates (62.5%, 15/24) harbored additional resistance to other antimicrobials (Fig. 5b), mainly sulfamethoxazole (45.8%, 11/24), ciprofloxacin (45.8%, 11/24) and chloramphenicol (37.5%, 9/24). Correspondingly, *sul1* and *sul2* (both 33.3%, 8/24) and *floR* (29.2%, 7/24) were among the most abundant antimicrobial resistance genes found (Fig. 5c). Resistance to ciprofloxacin was mainly associated with chromosomal point mutation *gyrA* p.S83L (29.2%, 7/24) or *qnrS1* (20.8%, 5/24). One isolate harboring the *parE* p.I355T mutation was not phenotypically associated with ciprofloxacin resistance. In one of three isolates, carriage of the *mph(A)* gene was not phenotypically associated with azithromycin resistance. Resistance to amikacin, colistin, meropenem or tigecycline was not detected.

Isolates showed overall high genetic diversity with presence of five different phylogroups (A, B1, B2, D, E) and 16 different MLST (Fig. 6). Across all farms and flocks, phylogroup B1 was most prevalent with 41.7% (10/24) of isolates, followed by A (25.0%, 6/24), D (25.0%, 6/24), B2 (4.2%, 1/24) and E (4.2%, 1/24). Among MLSTs, ST2179 and ST4035, both belonging to phylogroup B1, were most prevalent with three isolates each. Notably, different patterns were observed regarding the detection and genetic background of ESBL-/AmpC-producing *E. coli* depending on farm and flock; ranging from sporadic isolation at only one sampling time point to repeated detection of clonal isolates within a flock or across multiple flocks of a farm:

On farm 7 (flock 1, S4) and farm 9 (flock 1, S5), ESBL-/AmpC-producing isolates were observed at only one sampling time point across all flocks, meaning no distribution patterns could be identified. On both farms 13 and 8, only isolates of different phylogroups and STs were found within the same flock and in consecutive flocks.

Among the other farms and flocks however, five clusters of clonal ESBL-producing *E. coli* were observed (Table 4): On farm 4, three ST4035 isolates differing in 0-2 SNPs and sharing 98,8% of their genome were detected in flock 1 (S4) and flock 2 (S2 and S5). Additionally, two ST58 isolates differing in 5 SNPs were detected at S5 of both flock 1 and 2. Notably, neither of these clonal isolates were detected in flock 3, in which samples yielded no growth on MCA+CTX, or flock 4, in which only isolates belonging to ST362 (S4) were observed.

**Table 4:** Clonal clusters identified among 24 ESBL-producing *E. coli* isolates through cgMLST allele difference, shared reference genome and SNPs.

**Cluster**	**Isolates**	**SNPs**	**Shared Genome**	**cgMLST AD**	**MLST**	**Source**	**Hatchery**	**Phenotypical resistance**	**Resistance determinants**
1	23-EP00030-4	0	98.78	0	ST4035	F4-Fl1-S4	H3	AMP, CHL, FOT, GEN, SMX, TAZ, TMP	*aadA1; ant(2′')-Ia; blaCTX-M-15; dfrA36; floR; sul1; sul2*
	23-EP00091-4								
	23-EP00091-4	2		1		F4-Fl2-S2			
	23-EP00192-4								
	23-EP00192-4	2		1		F4-Fl2-S5			
	23-EP00030-4								

2	24-EP00133-4	0	99.81	0	ST5451	F14-Fl1-S1	H1	AMP, CIP, FOT, NAL, SMX, TAZ, TMP	*aadA22; aph(3′')-Ib; aph(6)-Id; blaSHV-12; dfrA1; lnu(F); qnrS1; sul1; sul2; gyrA p.S83L*
	24-EP00140-5					F14-Fl1-S2			*aadA1; aph(3′')-Ib; aph(6)-Id; blaSHV-12; dfrA1; lnu(F); qnrS1; sul1; sul2; gyrA p.S83L*

3	24-EP00122-4	2	99.50	1	ST10	F12-Fl4-S4	H4	AMP, CHL, CIP, FOT, SMX, TAZ	*aadA1; aadA2b; blaSHV-12; blaTEM-135c; cmlA1; lnu(F); qnrS1; sul3*
	24-EP00132-4					F12-Fl4-S5			

4	24-EP00128-4	0	99.88	0	ST2179	F2-Fl4-S3	H4	AMP, CHL, CIP, FOT, NAL, TAZ	*blaCTX-M-65; blaTEM-1B; floR; gyrA p.S83L; parC p.S80I*
	24-EP00144-4					F2-Fl4-S4			

5	23-EP00076-4	5	98,96	1	ST58	F4-Fl1-S5	H3	AMP, FOT, TAZ	*blaCTX-M-1*
	23-EP00192-5					F4-Fl2-S5			

F: farm, Fl: flock, S1: chicks upon delivery; S2: first two weeks of life; S3: first four weeks of life; S4: two weeks after first outdoor access; S5: age of slaughter.

On farm 12, clonal ST10 isolates (2 SNPs) were detected in subsequent sampling time points S4 and S5 of flock 4. All previous flocks and sampling time points had been negative for ESBL-/AmpC-producing *E. coli*. Similarly, the first two sampling time points in flock 1 of farm 14 showed growth of clonal ST5451 isolates (0 SNPs), but no growth of ESBL-/AmpC-producing *E. coli* was observed during the rest of the fattening period.

For the last cluster, clonal isolates belonging to ST2179 differing in 0 SNPs were found in subsequent sampling time points S3 and S4 in flock 4 of farm 2.

### Risk factors for occurrence of resistant isolates through mixed effects logistic regression

The null model and final model are available in Supplements 3.

As the random factor “farm” became redundant (intraclass correlation coefficient of 0) within the final model containing all fixed factors, it was excluded leaving only a random factor for farm*flock (adjusted ICC 0.053; conditional ICC 0.049). The final corrected model was significant (F (12, 683) = 3.427; *p* < 0.001) and demonstrated that the providing hatchery (F (5, 683) = 4.497; *p* < 0.001) and antimicrobial treatment (F (1, 683) = 7.714; *p* = 0.006) significantly explained the occurrence of resistant *E. coli*. No statistical significance was found for fattening type (F (2, 683) = 1.913; *p* = 0.148) or sampling time point (F (4, 683) = 1,362; *p* = 0.246).

Compared to broiler hatchery H2, isolates associated with broiler hatchery H5 were almost 11 (95% CI 3.821 – 30.865) times more likely to harbor resistance (*p* < 0.001). No significant difference in Odds Ratios was observed for other hatcheries. Antimicrobial treatment increased the odds of resistance compared to no treatment (OR = 14.968, CI 2.210 – 101.372) (Table 5).

**Table 5:** Regression coefficient, p-values and Odds Ratio (OR) with 95% confidence interval (CI) of fixed effects as estimated within generalized linear mixed models for commensal *E. coli* resistance towards at least one antimicrobial class.

	**Regression Coefficient**	**p-value**	**Odds Ratio**	**95% Confidence Interval Odds Ratio**	
				**Lower**	**Upper**
Intercept	**−3.233**	**<0.001**	**0.039**	**0.009**	**0.177**
slow-growing broiler	1.281	0.061	3.600	0.944	13.738
dual-purpose cockerels	0.455	0.331	1.576	0.629	3.952
male laying hybrids	0	-	-	-	-
S1 - chicks upon delivery	0.244	0.478	1.277	0.650	2.506
S2 - first two weeks of life	0.449	0.167	1.566	0.828	2.962
S3 - first four weeks of life	0.616	0.054	1.852	0.988	3.471
S4 - two weeks after outdoor access	0.052	0.881	1.053	0.535	2.071
S5 - age of slaughter	0	-	-	-	-
Hatchery H1	1.937	0.079	6.936	0.798	60.270
Hatchery H3	1.150	0.165	3.158	0.622	16.039
Hatchery H4	0.869	0.200	2.385	0.631	9.016
Hatchery H5	**2.385**	**<0.001**	**10.859**	**3.821**	**30.865**
Hatchery H6	0.974	0.119	2.648	0.778	9.012
Hatchery H2	0	-	-	-	-
Antimicrobial treatment	**2.706**	**0.006**	**14.968**	**2.210**	**101.372**
Without antimicrobial treatment	0	-	-	-	-

## Discussion

In this study, 51 flocks on 14 small to medium organic meat chicken farms were sampled longitudinally over a period of two years to examine the occurrence of antimicrobial resistant commensal and ESBL-/AmpC-producing *E. coli*. Further characterization of isolates exhibiting phenotypic resistance was conducted through whole genome sequencing to examine genetic resistance determinants and phylogenetic relationship.

### High percentage of susceptible commensal *E. coli* in all three fattening types

Throughout the fattening period, the percentage of susceptible isolates was high for slow-growing broiler (63.0-80.0%), male layer hybrids (76.9-97.6%) and dual-purpose cockerels (69.0-89.6%) alike. This aligns with previous resistance data from German organic meat chicken flocks assessed on farm in national AMR monitoring 2016, in which 71.0% (22/31) (vs. 13.3%; 40/301 from conventional flocks) of commensal *E. coli* were susceptible to all antibiotics tested. (Grobbel et al., 2022). A higher prevalence of susceptible *E. coli* isolates in the cecal contents of organic meat chickens compared to conventional meat chickens at the slaughterhouse has also been confirmed within Austria’s national monitoring program (average of 43.3% organic vs. 16.7% conventional in 2010-2014 and 2016) and in an Italian study comparing organic, antibiotic-free and conventional broiler flocks (8.1%; 22/272 vs. 7.9%; 23/290 vs. 3.1%; 9/292) (Much et al., 2019; Pesciaroli et al., 2020). Within the framework of national AMR surveillance, only one randomly selected isolate per epidemiological unit is included in antimicrobial susceptibility testing and sampling excludes smaller flocks to generate data representative for the overall population. Our results included three randomly selected isolates from each sample, to increase the likelihood of finding resistant strains and better depict the variety of the *E. coli* population at a given sampling time point. These differences in sampling design might explain comparatively higher rates of susceptible isolates in our dataset. Both low resistance rates in day-old chicks and low usage of antibiotics during rearing and fattening can be assumed to be a major contributing factor. Over the project period, only three out of 51 flocks were treated with tylosin, a macrolide, or amoxicillin, an aminopenicillin, respectively. Antimicrobial treatment was shown to significantly increase the odds of recovering resistant isolates in our study.

### Antimicrobial resistance rates over the course of fattening and recovery of non ESBL/AmpC MDR isolates differed between fattening types

Regardless of sampling time point and fattening type, most resistant commensal *E. coli* showed resistance to ampicillin (11.1%; 77/696), ciprofloxacin (9.9%; 69/696), tetracycline (9.5%; 66/696) or nalidixic acid (8.3%; 58/696). Resistance to sulfamethoxazole (6.8%; 47/696) and trimethoprim (4.9%; 34/696) were commonly detected as well, while resistance against chloramphenicol (2.3%, 16/696), azithromycin (0.4%, 3/696), gentamicin (1.1%, 8/696) and amikacin (0.3%, 2/696) was rare. These findings reflect the most common resistances in commensal *E. coli* detected within AMR surveillance in the European Union, where resistance to ampicillin, sulfamethoxazole, trimethoprim and tetracycline is most commonly observed in all livestock animals and rates of quinolone resistance are high among broiler (EFSA and ECDC, 2025). This is likely attributable to prolonged use of these antimicrobial classes in animal husbandry in the past shaping the resistome of the *E. coli* population, with resistance rates exhibiting a decline in recent years paralleled to decreased antimicrobial usage in the broiler sector for many European member states (Callens et al., 2018; EFSA and ECDC, 2025).

MDR isolates were obtained from all fattening types, but accounted for only a small proportion of 5.5% (38/696) overall (10.9%; 30/276 for slow-growing broiler, 1.9%; 4/210 for male layer hybrids, 1.9%; 4/210 for dual-purpose cockerels). A similar share of isolates was shown to be MDR in other studies on organic broilers from Germany (9.7%, 3/31) (Grobbel et al., 2022) and Austria (average of 22.7% CI 95% 10.3-43.8 in 2010-2014 and 2016) (Much et al., 2019), in which the same definition for multidrug-resistance (resistance to three or more antimicrobial classes) was used. In two other studies, multidrug-resistance was defined as resistance to five different antibiotics, resulting in 31.2% (85/272) MDR isolates from organic broiler flocks at slaughter in Italy (Pesciaroli et al., 2020), and as resistance to three or more different antibiotics, resulting in 35% (7/20) MDR isolates from an organic broiler farm in Poland (Lenart-Boron et al., 2016).

In male layer hybrids and dual-purpose cockerels, MDR isolates could not be detected within the first two weeks of life (S1-S2), hinting at a lower risk to be introduced with day-old chicks from the respective hatcheries. Notably, resistance of commensal *E. coli* to more than four antimicrobial classes only occurred in slow-growing broiler within our study and is attributable to clonal transmission of ST162 and ST57 in farm 3 introduced by day-old chicks from hatchery H5, as previously examined in detail (Korves et al., 2025).

### Detection of clonal *E. coli* isolates between flocks and farms suggest both introduction with day-old chicks and re-circulation of strains from the environment

A substantial number of clonal clusters was identified through whole genome sequencing. In addition to the anticipated recurrent isolation of clonal *E. coli* at varying sampling time points within one flock, a considerable number of clusters that spanned subsequent flocks within one farm could be observed. This suggests the circulation of certain *E. coli* strains in the environment, for example by surviving on surfaces in the chicken house after cleaning and disinfection, as has been demonstrated for conventional broiler flocks (Daehre et al., 2018; Leclercq et al., 2024; Robé et al., 2024) The possible survival of resistant strains in the soil of a shared outdoor enclosure must also be considered within the context of free-range farming.

However, several clonal clusters spanning different farms were identified as well, which cannot be attributed to a shared environment. In the majority of cases, an epidemiological link between farms could be established through the same hatchery providing day-old chicks. Additionally, the supplying hatchery was shown to be a highly significant factor for the recovery of resistant isolates in our statistical analysis. This emphasizes the importance of chick origin for the introduction of resistant strains into organic meat chicken flocks. Cluster II between slow-growing broiler farm 1 and dual-purpose cockerel farm 8 was the only cluster, where no link could be established from the information given, as day-old chicks were provided by two different hatcheries.

### Sporadic detection of genetically diverse ESBL-/AmpC-producing *E. coli*

Only a small percentage of samples tested positive for ESBL-/AmpC *E. coli* on selective medium; nonetheless, sporadic detection was observed in almost two thirds of the farms and 27.5% of flocks. Notably, only one flock tested positive at S1, although day-old chicks are often discussed as a possible vector for introducing ESBL-/AmpC-producing and otherwise antimicrobial resistant *E. coli* into conventional flocks. However, this flock was unique in being the only flock rearing three-day-old Dekalb White chicks for S1. A longitudinal study of two consecutive organic broiler flocks in the Netherlands showed that day-old chicks sampled directly upon arrival were already positive for ESBL-/AmpC-producing *E. coli* of phylogroup A1 at a lower prevalence in the first flock, but negative in the second flock (Huijbers et al., 2016). Interestingly, both flocks examined by Huijbers et al. (2016) reached their peak regarding intra-flock prevalence at day three, after chicks of the second flock supposedly have been recolonized by strains persisting in the broiler house.

Sporadic detection of ESBL-producing *E. coli* in our study might also be related to overall reduced load within the gut of organic broiler compared to antibiotic-free and conventional chicken (Tofani et al., 2022). A possible connection between a reduced load of ESBL-producing *E. coli* in the gut of organic broiler due to less contact to manure and litter material in the context of mandatory outdoor access has been discussed by Musa et al. (2020). An experimental study by Chuppava et al. (2019) demonstrated, that animal-to-animal contact due to crowding because of attractive areas or overall higher stocking density was even more important for the prevalence of MDR *E. coli* in broiler, than the contact to manure alone. In our study, sampling time points S1 to S3 mark the timeframe of chick rearing without access to the outdoor run, where the most intensive contact to the litter and crowding effects around attractive areas within the chicken house (feed and water lines, enrichment) can be assumed. Most positive flocks for ESBL-/AmpC-producing *E. coli* were identified at sampling time points S4 and S5. At this point, the birds were both older, had outdoor access and had been exposed to possible vectors for ESBL-/AmpC-producing *E. coli* from their environment, such as water, feed, farm equipment, farm personnel, rodents and insects for a longer period of time. Given that in farm 4, the same clonal isolates of different ST4035 and ST58 were repeatedly detected in subsequent flocks, the re-circulation of these strains from the farm environment seems plausible (Leclercq et al., 2024; Robé et al., 2024). This is emphasized by the fact that the same hatchery also supplied dual-purpose chicks to farm 6 and farm 8, yet ESBL-producing ST4035 and ST58 were only isolated from farm 4.

It could thus be hypothesized, that I) day-old chicks from our study were truly negative for ESBL-/AmpC-producing *E. coli* upon arriving at farm and were colonized at later stages of the fattening period with strains from their housing environment, or that II) due to low initial colonization of day-old chicks, detection using our methodology at this age was unsuccessful and became more reliable in older chicks with suspected higher intra-flock prevalence.

The variety of strains detected within our study could therefore reflect both acquisition within a hatchery as well as additional strains circulating within the chicken’s individual environment.

### Study limitations

According to the Federal Statistical Office of Germany, 70% of all organic meat chicken are reared in farms with more than 10,000 birds per farm. However, these are only 9.8% of all German farms rearing organic meat chicken. Most of the farms are small to medium sized farms like the farms included in our project (Statistisches Bundesamt (Destatis), 2024). The results therefore can be considered representative for the farm sizes and variety of chicken fattening types reared in the organic sector in Germany. The study design was limited to sampling the housing compartment on the respective farms and no additional samples were taken from the surrounding area, supplying hatcheries or breeder flocks. Nevertheless, by sampling chick transport boxes and carefully tracking chick origin through the provided metadata, possible sources of resistant strains were investigated as accurately as possible.

## Conclusion

Our study was the first to compare antimicrobial resistance between organically raised slow-growing broiler, male layer hybrids and dual-purpose cockerels in Germany.

The majority of isolates from all age groups and fattening types was phenotypically susceptible to all antimicrobials tested. ESBL-/AmpC- producing *E. coli* were only sporadically detected through selective isolation in roughly a quarter of all flocks sampled. No significant differences in the recovery of resistant commensal isolates could be observed between fattening types or depending on sampling time point. Clonal clusters of resistant strains were discovered not only within a flock, but also across subsequent flocks and even different farms through whole genome sequencing. As the providing hatchery was shown to have a highly significant influence on the recovery of resistant commensal isolates, introduction with day-old chicks explains the recovery of resistant and multidrug-resistant commensal *E. coli*.

Further studies are required to improve the understanding of transmission routes of resistant and ESBL-/AmpC-producing *E. coli* within organic meat chicken farms and should encompass sampling of larger flocks and farms, the housing environment and outdoor enclosures.

## Data availability

Assembled genome sequences were provided at the National Center for Biotechnology Information (NCBI) under Bioproject PRJNA1290230.

## Ethics statement

The project was evaluated and approved by the Central Ethics Committee of the University of Kassel (Case number: zEK-62) and the Department of Veterinary Affairs and Consumer Protection of the Regional Council of Kassel (Case number: RPKS - 23-19 c 16/15-2018/5).

## Funding

The project was funded by the Federal Ministry of Agriculture, Food and Regional Identity (BMLEH) based on a decision of the parliament of the Federal Republic of Germany via the Federal Office for Agriculture and Food (BLE) under the Federal Program for Organic Farming (FKZ: 2821OE035). The funders were not involved in the study design, collection, analysis, interpretation of data, the writing of this article or the decision to submit it for publication.

## Declaration of interests

The authors declare that they have no known competing financial interests or personal relationships that could have appeared to influence the work reported in this paper.

## CRediT authorship contribution statement

**Anna Maria Korves-Wilm:** Writing – original draft, Visualization, Investigation, Formal analysis, Data curation. **Mirjam Grobbel:** Writing – review & editing, Supervision. **Bernd-Alois Tenhagen:** Writing – review & editing, Supervision, Project administration, Conceptualization.

## Disclosures

The authors have declared that no conflict of interest exists.

## Appendix Supplementary materials

Image, application 1

Image, application 2

Image, application 3
