# Supplementary material for: Antimicrobial resistance of commensal and extended-spectrum ß-lactamase/AmpC-producing Escherichia coli in organic meat chicken farms
Source: Poult Sci. 2026 Jan 30;105(4):106559. doi: 10.1016/j.psj.2026.106559 (PMC12925275; doi:10.1016/j.psj.2026.106559)
Supplement: Supplementary file 2 [file mmc2.pdf]

Supplementary Materials 2: Antimicrobial resistance of commensal and extended-spectrum ß-lactamase/AmpC-producing *Escherichia coli* in organic meat chicken farms

Anna Maria Korves-Wilm*[1], Mirjam Grobbel*, Bernd-Alois Tenhagen*

Authors Affiliations:

* Department Biological Safety, German Federal Institute for Risk Assessment, Berlin, Germany

Supplementary Table 1: Hatcheries providing chicks for project farms with chicken type and breed used, number of flocks provided per farm and organic certification status

Hatchery ID	Certified organic?	EU member state	Chicken type	No. of flocks provided	Farm ID
**H1**	yes	DE	M	1	14
**H2**	yes	DE	B	4	1
			B	1	3
			B	4	7
			B	4	10
**H3**	yes	DE	D	4	4
			D	4	6*
**H4**	yes	DE	B	2	2
			M	2	5
			D	4	9*
			M	4	11
			M	4	12
			M	4	13
**H5**	yes	DE	B	3	3
**H6**	yes	NL	B	2	2

B = slow-growing broiler
M = male layer hybrids
D = dual-purpose cockerels

DE = Germany
NL = Netherlands

* farm 6 and farm 9 reared chickens for farm 8

Supplementary Table 2: Putative clusters of commensal E. coli isolates through cgMLST allele difference, shared reference genome and SNPs

Cluster	Isolates	SNPs	Shared Genome	cgMLST AD	MLST	Source	Hatchery	Phenotypical resistance	Resistance determinants
1	23-EP00078-2<br>24-EP00033-1	137	98,44	0	ST2223	F3-Fl2-S2<br>F3-Fl4-S2	H5<br>H2	CIP, NAL	*qnrB19*
2	23-EP00112-2<br>23-EP00204-3	20	94,43	5	ST155	F5-Fl1-S5<br>F10-Fl3-S5	H4<br>H2	AMP, AZI<br>AZI, TET	*blaTEM-1B; mph(A)*<br>*mph(A); tet(A)*
3	24-EP00068-2<br>24-EP00138-2	671	96,37	5	ST17744	F9-Fl3-S3<br>F9-Fl4-S4	H4	TET, TMP<br>TET	*aadA1; dfrA1; sat2; tet(A)*<br>*tet(A)*
4	24-EP00026-3<br>24-EP00122-2	76	98,28	10	ST10	F12-Fl3-S3<br>F12-Fl4-S4	H4	CIP, NAL	*gyrA p.S83L*
5	24-EP00036-1<br>24-EP00047-2	172	98,19	4	ST155	F9-Fl2-S2<br>S9-Fl3-S1	H4	AMP	*blaTEM-1C*
	23-EP00142-1<br>23-EP00154-3	306		6		F3-Fl3-S2	H5		
6	23-EP00154-3<br>23-EP00214-3	306	98,48	7	13322	F2-Fl2-S4	H6	CIP, NAL, TET	*tet(B); gyrA p.S83L; gyrA p.D87N; parC p.A56T; parC p.S80I*
	23-EP00214-3<br>23-EP00142-1	4		1		F3-Fl3-S5	H5		

	23-EP00102-1 23-EP00170-3	8		7		F11-Fl2-S3		AMP, SMX, TET, TMP	*aadA1; aadA5; aph(3'')-Ib; aph(6)-Id; blaTEM-1B; dfrA1; dfrA17; lnu(G); sul1; sul2; tet(A)*
	23-EP00102-1 24-EP00026-2	498		5		F12-Fl3-S3		AMP, SMX, TMP	*aadA1; aadA5; blaTEM-1B; dfrA17; lnu(G); sul2*
7	23-EP00102-1 24-EP00056-1	10	96,84	6	ST155		H4		
	23-EP00170-3 24-EP00026-2	502		7		F13-Fl3-S3		AMP, SMX, TET, TMP	*aadA1; aadA5; aph(3'')-Ib; aph(6)-Id; blaTEM-1B; dfrA1; dfrA17; lnu(G); sul1; sul2; tet(A)*
	23-EP00170-3 24-EP00056-1	6		3		F2-Fl3-S5		AMP, SMX, TET, TMP	*aadA1; aadA5; aph(3'')-Ib; aph(6)-Id; blaTEM-1B; dfrA1; dfrA17; lnu(G); sul1; sul2; tet(A)*
	24-EP00026-2 24-EP00056-1	504		6					

Clusters were not classified as clonal due to >20 SNP identified between isolates
