# Supplementary material for: Antimicrobial resistance of commensal and extended-spectrum ß-lactamase/AmpC-producing Escherichia coli in organic meat chicken farms
Source: Poult Sci. 2026 Jan 30;105(4):106559. doi: 10.1016/j.psj.2026.106559 (PMC12925275; doi:10.1016/j.psj.2026.106559)
Supplement: Supplementary file 3 [file mmc3.pdf]

Supplementary Materials 3: Antimicrobial resistance of commensal and extended-spectrum ß-lactamase/AmpC-producing *Escherichia coli* in organic meat chicken farms

Anna Maria Korves-Wilm*[1], Mirjam Grobbel*, Bernd-Alois Tenhagen*

Authors Affiliations:

* Department Biological Safety, German Federal Institute for Risk Assessment, Berlin, Germany

Generalized linear mixed models – Model summary

Null model: intercept only model, includes random intercept for farm and farm*flock

GLMM 1: full model containing fixed effects and random intercept for farm and farm * flock

GLMM 2: full model containing fixed effects and random intercept for farm * flock

		Nullmodell	GLMM 1	GLMM 2
**Case Processing Summary**	**included**	696	696	696
	**excluded**	0	0	0
	**total**	696	696	696
**Model Summary**	**target**	resistance_bin	resistance_bin	resistance_bin
	**Probability distribution**	binominal	binominal	binominal
	**link function**	logit	logit	logit
**Model Fitness**	**Akaike corrected (AIC)**	3269.208	3331.443	3329.431
	**Bayesian (BIC)**	3278.279	3340.478	3333.952
	**-2 Log-Likelihood**	3265.191	3327.425	3327.425
	**Pseudo-R^2 measure (marginal)**	0.000	0.083	0.083
	**Pseudo-R^2 measure (conditional)**	0.100	0.132	0.132
**Intraclass Correlation Coefficients**	**Overall ICC (adjusted)**	0.100	0.053	0.053
	**Overall ICC (conditional)**	0.100	0.049	0.049
	**Farm ICC (adjusted)**	0.048	0.000	/
	**Farm ICC (conditional)**	0.048	0.000	/
	**Farm*flock ICC (adjusted)**	0.052	0.053	/
	**Farm*flock ICC (conditional)**	0.052	0.049	/

As ICC for farm was 0 in GLMM 1 and did not result in a stable model, GLMM 2 was chosen as the final model and showed a better fit based on AICc and BIC. Fixed effects and fixed coefficients result tables show no differences between GLMM1 and GLMM2.

Generalized linear mixed models – Fixed effects

GLMM 1

Source	F	df1	df2	Sig.
Corrected Model	3.427	12	683	< 0.001
Fattening type	1.913	2	683	0.148
Sampling time point	1.362	4	683	0.246
Hatchery	4.497	5	683	< 0.001
AB treatment	7.714	1	683	0.006

GLMM 2

Source	F	df1	df2	Sig.
Corrected Model	3.427	12	683	< 0.001
Fattening type	1.913	2	683	0.148
Sampling time point	1.362	4	683	0.246
Hatchery	4.497	5	683	< 0.001
AB treatment	7.714	1	683	0.006

Generalized linear mixed models – Fixed coefficients

GLMM 1

**Model Term**	**Coefficient**	**Std. Error**	**t**	**Sig.**	**95% Confidence Interval**		**Exp(Coefficient)**	**95% Confidence Interval for Exp(Coefficient)**	
					**Lower**	**Upper**		**Lower**	**Upper**
Intercept	-3.233	0.7660	-4.221	0.000	-4.737	-1.729	0.039	0.009	0.177
Slow-growing broiler	1.281	0.6820	1.878	0.061	-0.058	2.620	3.600	0.944	13.738
Dual-purpose males	0.455	0.4681	0.972	0.331	-0.464	1.374	1.576	0.629	3.952
Male laying hybrids	0[b]								
Sampling time point S1	0.244	0.3436	0.711	0.478	-0.430	0.919	1.277	0.650	2.506
Sampling time point S2	0.449	0.3245	1.383	0.167	-0.188	1.086	1.566	0.828	2.962
Sampling time point S3	0.616	0.3199	1.927	0.054	-0.012	1.244	1.852	0.988	3.471
Sampling time point S4	0.052	0.3445	0.150	0.881	-0.625	0.728	1.053	0.535	2.071
Sampling time point S5	0[b]								
Hatchery H1	1.937	1.1012	1.759	0.079	-0.226	4.099	6.936	0.798	60.270
Hatchery H3	0.974	0.6238	1.561	0.119	-0.251	2.199	2.648	0.778	9.012
Hatchery H4	1.150	0.8277	1.389	0.165	-0.475	2.775	3.158	0.622	16.039
Hatchery H5	0.869	0.6773	1.283	0.200	-0.461	2.199	2.385	0.631	9.016
Hatchery H6	2.385	0.5320	4.483	0.000	1.340	3.430	10.859	3.821	30.865
Hatchery H2	0[b]								
With AB treatment	2.706	0.9743	2.777	0.006	0.793	4.619	14.968	2.210	101.372
Without AB treatment	0[b]								

GLMM 2

Model Term	Coefficient	Std. Error	t	Sig.	95% Confidence Interval		Exp(Coefficient)	95% Confidence Interval for Exp(Coefficient)	
					Lower	Upper		Lower	Upper
Intercept	-3.233	0.7660	-4.221	0.000	-4.737	-1.729	0.039	0.009	0.177
Slow-growing broiler	1.281	0.6820	1.878	0.061	-0.058	2.620	3.600	0.944	13.738
Dual-purpose males	0.455	0.4681	0.972	0.331	-0.464	1.374	1.576	0.629	3.952
Male laying hybrids	0^b								
Sampling time point S1	0.244	0.3436	0.711	0.478	-0.430	0.919	1.277	0.650	2.506
Sampling time point S2	0.449	0.3245	1.383	0.167	-0.188	1.086	1.566	0.828	2.962
Sampling time point S3	0.616	0.3199	1.927	0.054	-0.012	1.244	1.852	0.988	3.471
Sampling time point S4	0.052	0.3445	0.150	0.881	-0.625	0.728	1.053	0.535	2.071
Sampling time point S5	0^b								
Hatchery H1	1.937	1.1012	1.759	0.079	-0.226	4.099	6.936	0.798	60.270
Hatchery H3	0.974	0.6238	1.561	0.119	-0.251	2.199	2.648	0.778	9.012
Hatchery H4	1.150	0.8277	1.389	0.165	-0.475	2.775	3.158	0.622	16.039
Hatchery H5	0.869	0.6773	1.283	0.200	-0.461	2.199	2.385	0.631	9.016
Hatchery H6	2.385	0.5320	4.483	0.000	1.340	3.430	10.859	3.821	30.865
Hatchery H2	0^b								
With AB treatment	2.706	0.9743	2.777	0.006	0.793	4.619	14.968	2.210	101.372
Without AB treatment	0^b								
